# Effects of Exogenous Chlorinated Amino Acetic Acid on Cadmium and Mineral Elements in Rice Seedlings

**DOI:** 10.3390/toxics11010071

**Published:** 2023-01-12

**Authors:** Shuangyue Liu, Lin Fu, Changbo Zhang, Jiawei Deng, Weijie Xue, Yun Deng

**Affiliations:** 1 Key Laboratory of Original Agro-Environmental Pollution Prevention and Control, Agro-Environmental Protection Institute, Ministry of Agriculture and Rural Affairs, Tianjin 300191, China; 2 School of Environment and Civil Engineering, Jiangnan University, Wuxi 214122, China

**Keywords:** rice, cadmium, amino acids, chlorinated amino acetic acid, mineral elements, plant stress

## Abstract

To explore the effect of exogenous application of chlorinated amino acetic acid on cadmium (Cd) transport characteristics in rice seedlings, X24 and Z35 rice were taken as the research objects to carry out hydroponics experiments, and the changes of Cd content in rice seedlings, rice mineral elements and amino acid content in rice were analyzed. The results showed that exogenous application of 1.2 mmol·L^−1^ chlorinated amino acetic acid inhibited cadmium in shoots and roots of rice seedlings; Cd content in shoots and roots were reduced by up to 62.19% and 45.61%, respectively. The majority of cadmium was in the cell wall of shoots and roots; this decreased with the increase of the concentration of chlorinated acetic acid. In addition, the Mn content in shoots and Ca content in roots of rice seedlings increased significantly after the application of chlorinated amino acetic acid. The results of amino acid analysis showed that the contents of aspartic acid, glutamic acid and cystine in rice seedlings were increased. These results indicate that exogenous application of chlorinated amino acetic acid is beneficial to the synthesis of aspartic acid, glutamic acid and cysteine in rice seedlings, increases the content of Mn in shoots and Ca in roots of rice seedlings, and significantly alleviates cadmium stress in seedlings. This provides a theoretical basis for the development of an environmentally friendly Cd-lowering foliar fertilizer for rice.

## 1. Introduction

In recent years, the rapid increase of human activities has led to the destruction of the soil environment; heavy metal pollution often threatens soil quality and human health [1]. Monitoring of soil heavy metal content shows that the types of heavy metal pollution in farmland soil are constantly increasing, the area is constantly expanding, and the degree is constantly intensifying [2]. Soil heavy metal pollution is characterized by strong concealment, poor self-purification ability, and long risk accumulation time. While limiting the absorption of soil nutrients by crops, heavy metals can accumulate in the soil for a long period [3]. Cadmium is a heavy metal with strong toxicity, strong activity in soil, and easy absorption and enrichment by crops. It is highly transferable in soil–plant systems, and it is easily absorbed by plant roots and transported to the aboveground parts [4].

As one of the three grain crops in the world, rice is the grain crop with the largest planted area and a high yield per unit area. Cadmium, as the main heavy metal pollutant in rice, is easily enriched in rice and poses a threat to human health through the food chain. Cadmium is usually absorbed by the roots of rice and transported to the overground part after loading in xylem and phloem, which negatively impedes nutrient absorption and the normal physiological transport processes of rice [5,6,7]. When cadmium exceeds 0.2 mg/kg, it will have a toxic effect on rice cells, causing the rice stressed by cadmium to show chlorosis, necrosis and other toxic symptoms [8]. Under cadmium stress, many transporters are involved in cadmium uptake, transport, chelation and detoxification. Most metal ions enter plant roots through specific ionic carriers or channels. Since cadmium is not an essential element for plant growth, it usually enters plant somatic cells through the transport system of divalent cations such as Zn^2+^, Fe^2+^, Ca^2+^ and Mn^2+^ [9,10]. A high concentration of cadmium may hinder plant protein metabolism by changing plant physiological function and inhibiting synthetic activity, interfere with the coordination mechanism between plant basic elements, and induce excessive production of reactive oxygen species, which will directly or indirectly destroy plant molecular function and even lead to plant death in severe cases [11,12]. Studies have shown that a variety of exogenous additives can significantly alleviate cadmium stress in rice seedlings [13,14,15].

**Figure 1:**
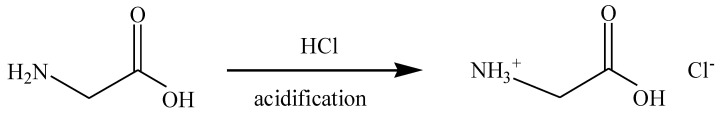
The reaction diagram of chlorinated amino acetic acid formation.

Ionic liquid is a kind of salt composed of organic cations and anions. It has a good leaching effect on heavy metal cadmium in soil, with a removal rate of cadmium up to 55.4% [16,17]. Glycin, as an important amino acid molecule in plants, is involved in the regulation of plant nitrogen metabolism and plays an important fundamental role in nerve signal transmission, maintenance of cell REDOX balance, regulation of ion channel activity and sensitivity to cell stress response [18,19]. Studies have shown that the toxicity of cadmium pollution in paddy fields can be mitigated by triggering amino acid biosynthesis of L-lysine, glycine or glutamine in rice roots and the subsequent accumulation of amino acids in rhizosphere soil [20]. Chlorinated amino acetic acid is a glycine salt formed by acidification, with glycine as a precursor and water as a solvent (Figure 1). It is a green ionic liquid that is easily soluble in water and highly stable. In this paper, the regulation mechanism of chlorinated amino acetic acid on cadmium accumulation and transport in rice seedlings was explored by adding more water-soluble ionic liquid chlorinated acetic acid at the seedling stage, which can provide data support for the development of environmentally protective Cd-lowering leaf fertilizer for rice, reducing the harm of Cd to rice and human beings and thus being of great significance for ensuring food security.

## 2. Materials and Methods

### 2.1. Experimental Materials and Locations

Rice seeds from indica rice (*Oryza sativa L.*) cultivar X24 and Z35 were selected as experimental plants. The hydroponic experiment was carried out in the artificial climate room of the Agro-Environmental Protection Institute, Ministry of Agriculture and Rural Affairs, in Tianjin.

### 2.2. Cultivation and Processing Methods

The test seeds were soaked in 5% H_2_O_2_ for 30 min, washed with deionized water, and spread evenly in the seedling tray; deionized water was added to cover the seeds, and then they were germinated in a constant temperature incubator at 25 °C for 48 h under dark conditions. After the seeds took root and sprouted, they were transferred to the artificial climate room for cultivation. When the rice seedlings grew to two leaves and one heart, the rice seedlings were moved to an 8 L incubator containing 1/10 Hoagland nutrient solution and continued to be cultivated in the artificial climate room. Condition parameters of the artificial climate chamber were as follows: 16 h of daylight, 8 h of darkness; light intensity: 105 μmol·m^−2^·s^−1^; day and night temperature: 28 °C/25 °C; relative humidity: 60%. When the rice seedlings grew to the three-leaf and one heart stage, uniform rice seedlings were selected, and the treatment was applied for 7 days. The treatment solution was changed every 2 days.

This experiment consisted of 5 treatments; each treatment was repeated 3 times, 2.7 μmol·L^−1^ CdCl_2_ was added, and 0, 0.2, 0.5, 0.8 and 1.2 mmol·L^−1^ chlorinated amino acetic acid was applied, respectively. The pH of the treatment group solution was adjusted to 5.6–6.5 with KOH and HNO_3_.

After 7 days of treatment, rice seedlings were soaked and cleaned with 5 mmol·L^−1^ CaCl_2_ for 30 min, and then the roots were repeatedly washed with deionized water. Fresh rice seedling samples were used for the determination of subcellular Cd distribution, while dry rice seedling samples were used for the determination of Cd, mineral elements and total amino acids.

### 2.3. Determination of Cd, K, Ca, Mg, Fe, Mn and Zn Concentrations

A total of 0.50 g of grain and 0.25 g of other parts were accurately weighed and poured into the sterilization tube; 7.0 mL MOS nitric acid was added and placed for 5 h. The samples were digested at 110 °C for 2.5 h on a ED54 electrothermal digestion apparatus. After cooling, 1.0 mL of high-grade pure hydrogen peroxide was added. The samples were digested at 110 °C for 1.5 h, and then the acid was removed by heating the apparatus to 170 °C. The sterilizing tube was rinsed with deionized water, placed in a 25 mL volumetric flask, and finally poured it into a 25 mL square flask for storage. The concentrations of cadmium, K, Ca, Mg, Fe, Mn and Zn in different plant samples were determined by inductively coupled plasma mass spectrometry (ICP-MS, Agilent 7500a, CA, USA).

### 2.4. Determination of Subcellular Cadmium Content

Then, 1.0 g of shoot and 0.5 g of root of rice seedlings were weighed and homogenized in extraction buffer (50 mmol·L^−1^ Tris-HCl, 250 mmol·L^−1^ sucrose and 1.0 mmol·L^−1^ dithioerythritol) using a homogenizer. The homogenate was passed through a 100-mesh nylon sieve, and the precipitated fraction of the residue and filtrate was collected after centrifugation at 3500 rpm·min^−1^ for 5 min, which was the cell wall (F1) fraction. The supernatant was poured into a new centrifuge tube and centrifuged at 4 °C at a speed of 15,000 rpm·min^−1^ for 40 min. The precipitation was the organelle (F2) component, and the supernatant was the cytoplasm (F3) component.

### 2.5. Determination of Total Amino Acids

Then, 15.0 mL 6 mol·L^−1^ HCl was added to the test tube. After cooling the test tube for 5 min in an ice bath, the test tube was filled with high-purity nitrogen, and the lid was tightened quickly. After that, the test tube was placed in a constant temperature air-drying oven at 110 °C for 22 h. Then, the tube was taken out, and the acid solution was cooled to room temperature. After filtration, the volume was fixed to 50.0 mL to obtain the subtracting solution. After taking a constant volume, 1.0 mL of the solution was blown dry with nitrogen in a water bath with 50 °C, and then 1.0 mL of deionized water was added for another repeat. Finally, the solution was dissolved in 2.0 mL (pH = 2.2) sodium citrate buffer, filtered through a 0.22 μm filter membrane, and placed in a 2.0 mL injection bottle. A total of 20.0 μL of the acid lysate was added to 50.0 μL 0.4 N borate buffer (pH = 10.2), and the solution was thoroughly mixed for 1 min. Then, 10.0 μL OPA reagent was added, and the derivatives were mixed thoroughly for 3 min. Amino acids were determined by high performance liquid chromatograph (Agilent Technologies, 1200 series, USA).

### 2.6. Data Statistics and Analysis

All the experimental data were the mean values obtained in 3 replicates, and all the data were expressed as the mean ± standard deviation. One-way analysis of variance (ANOVA) and the Duncan test were used for multiple comparisons, and *p* < 0.05 was used to evaluate the differences between treatments. Origin 2018 and Microsoft Excel 2021 were used for data processing and mapping. SPSS 26.0 software was used for statistical significance analysis.

## 3. Results

### 3.1. Effects of Chlorinated Amino Acetic Acid on Cadmium Content in Rice Seedlings

**Figure 2:**
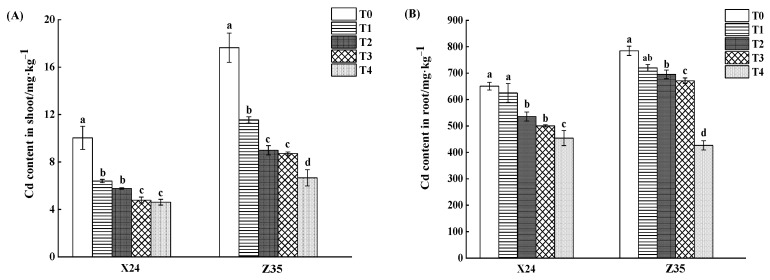
Effects of chlorinated amino acetic acid on cadmium content in shoots (**A**) and roots (**B**) of rice seedlings. T0, T1, T2, T3 and T4 respectively represent the application of 0, 0.2, 0.5, 0.8 and 1.2 mmol·L^−1^ chlorinated amino acetic acid treatment. Different lowercase letters indicate significant differences between treatments (*p* < 0.05).

After the application of 0.2–1.2 mmol·L^−1^ chlorinated amino acetic acid, the Cd content in the shoots and roots of rice seedlings significantly decreased, and the accumulation of Cd in shoots was much lower than that in roots (Figure 2). Compared with the control, 1.2 mmol·L^−1^ chlorinated amino acetic acid had the highest inhibition efficiency on cadmium in X24 and Z35, and the content of Cd in X24 shoots decreased from 10.03 mg·kg^−1^ to 4.61 mg·kg^−1^. The Cd content in shoots of Z35 seedlings decreased from 17.64 mg·kg^−1^ to 6.67 mg·kg^−1^, decreasing by 54.04% and 62.19%, respectively. The root Cd content of X24 seedlings decreased from 650.60 mg·kg^−1^ to 454.17 mg·kg^1^, and the root Cd content of Z35 seedlings decreased from 784.36 mg·kg^−1^ to 426.61 mg·kg^−1^, with decreases of 30.19% and 45.61%, respectively.

### 3.2. Effects of Chlorinated Amino Acetic Acid on Cadmium Content in Subcellular of Rice Seedlings

**Figure 3:**
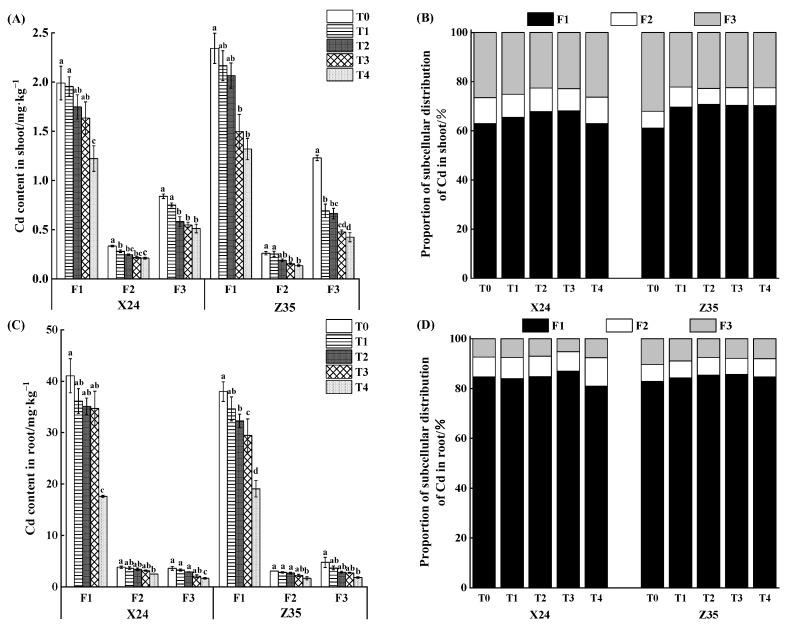
Effects of chlorinated amino acetic acid on cadmium concentration in shoot (**A**,**B**) and root (**C**,**D**) subcellular fractions of rice seedlings. T0, T1, T2, T3 and T4 respectively represent the application of 0, 0.2, 0.5, 0.8 and 1.2 mmol·L^−1^ chlorinated amino acetic acid treatment. Different lowercase letters indicate significant differences between treatments (*p* < 0.05).

Under cadmium stress, cadmium distribution in different subcellular fractions in shoots and roots of rice seedlings was different. Figure 3 shows the content and distribution of cadmium in shoots and roots of X24 and Z35 after different concentrations of chlorinated amino acetic acid were applied under cadmium stress. Most cadmium in shoots and roots exists in the cell wall (F1); the organelle (F2) had the lowest amount, while the remainder was in cytosol (F3). Compared with the control, the cadmium content in shoot and root subcellular fractions of X24 and Z35 decreased after the application of chlorinated acetic acid. When 1.2 mmol·L^−1^ chloroacetic acid was applied, the cadmium content in shoot and root cell walls of rice seedlings was significantly decreased, with the decrease of cadmium content in shoots and roots of X24 and Z35 being 38.56% and 43.69%, respectively, and 57.12% and 49.78%, respectively. Under Cd stress, the total content of Cd in each subcellular fraction decreased after the application of chlorinated amino acetic acid; the proportion of Cd in the cell wall fraction of rice seedlings showed an increasing trend, while the proportion of Cd in the cell fluid fraction showed a decreasing trend (Figure 3B,D).

### 3.3. Effect of Chlorinated Amino Acetic Acid on Content of Mineral Elements in Rice Seedlings

In this experiment, the contents of mineral elements (K, Mg, Ca, Fe, Mn, Zn) in rice seedlings were determined (Table 1 and Table 2). The content of mineral elements in Z35 was generally higher than that in X24, and there were significant differences in the content of mineral elements in the shoots and roots of rice. As shown in Table 1 and Table 2, the content of K was the highest in the aboveground part of rice seedlings, followed by the content of Mg and Ca, which was much higher than that of Fe, Mn and Zn. The content of K in the roots of rice seedlings was the highest, followed by Fe and Zn. Compared with the control, the contents of K, Mg, Ca, Fe and Zn in the aboveground parts of X24 and Z35 were not significantly affected by exogenous application of 1.2 mmol·L^−1^ chlorinated amino acetic acid, while the contents of Mn were increased by 34.78% and 26.24%, respectively. The contents of Mg and Zn in X24 and Z35 roots were not significantly affected, while the contents of Ca were increased by 11.64% and 53.39%, respectively.

### 3.4. Effect of Chlorinated Amino Acetic Acid on Total Amino Acid Content of Rice Seedlings

**Figure 4:**
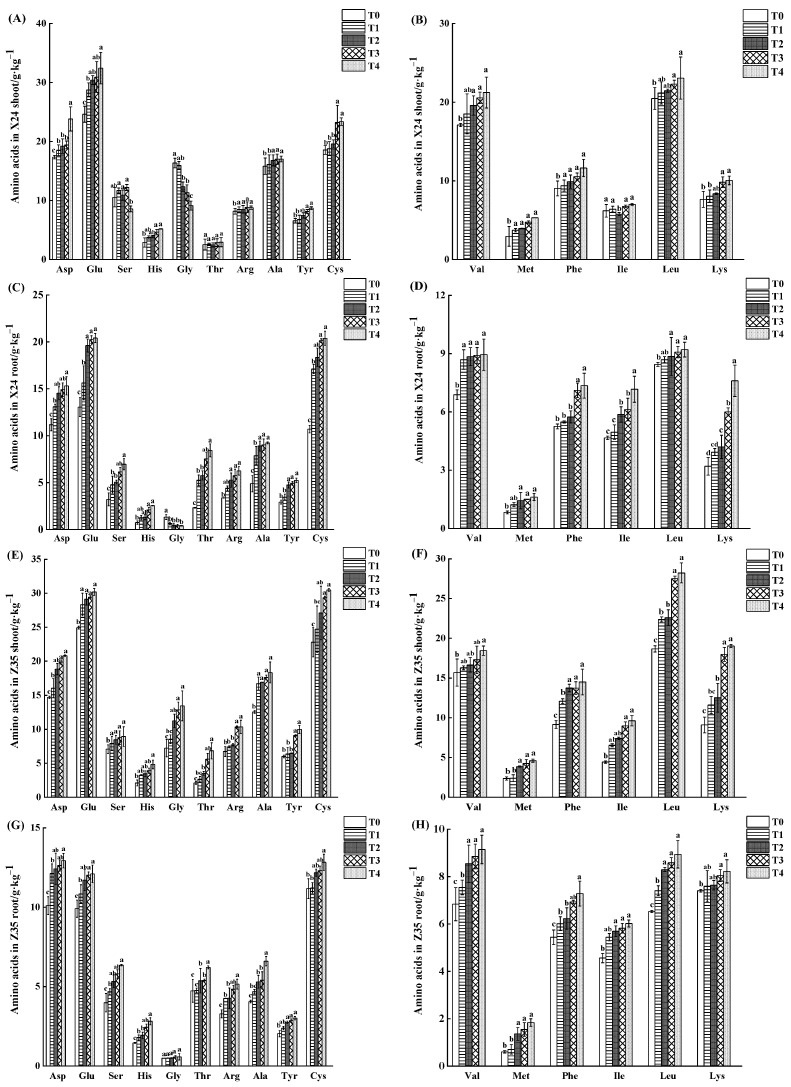
Effect of chlorinated amino acid on total amino acid content in shoots (**A**,**B**,**E**,**F**) and roots (**C**,**D**,**G**,**H**) of rice seedlings. T0, T1, T2, T3 and T4 respectively represent the application of 0, 0.2, 0.5, 0.8 and 1.2 mmol·L^−1^ chlorinated amino acetic acid treatment. Different lowercase letters indicate significant differences between treatments (*p* < 0.05).

Under cadmium stress, the content of amino acids in rice shoots and roots was generally increased by the application of chlorinated acetic acid. The amino acids in shoots and roots of rice seedlings were affected more by a high concentration of chlorinated amino acid than by a low concentration. Among them, aspartic acid (Asp), glutamic acid (Glu), serine (Ser), histidine (His), glycine (Gly), threonine (Thr), arginine (Arg), alanine (Ala), tyrosine (Tyr) and cystine (Cys) are non-essential amino acids; Valine (Val), methionine (Met), phenylalanine (Phe), isoleucine (Ile), leucine (Leu), and lysine (Lys) are essential amino acids (Figure 4). After the application of 1.2 mmol·L^−1^ chlorinated amino acetic acid, glutamate was the most abundant amino acid in X24 shoots and roots, with a content of 32.44 g·kg^−1^ and 20.41 g·kg^−1^, respectively, followed by aspartic acid and cystine; threonine had the lowest accumulation. Leucine had the highest content of essential amino acids, at 23.06 g·kg^−1^ and 9.21 g·kg^−1^, respectively. The content of glutamate in shoots of Z35 was the highest, at 30.19 g·kg^−1^. The highest content of aspartic acid was 12.93 g·kg^−1^ in the root of Z35.

Among the 16 amino acids, glutamic acid and aspartic acid were more sensitive to cadmium stress than the other amino acids and showed a trend of significant increase with the decrease of cadmium content in shoots and roots of rice seedlings. The contents of aspartate, glutamic acid, cystine and valine in X24 shoots were significantly increased by 37.35%, 31.76%, 25.85% and 24.17%, respectively, after the application of 1.2 mmol·L^−1^ chlorinated amino acetic acid. The glycine content was significantly reduced by 44.19%. The contents of aspartic acid, glutamic acid, cystine and valine in X24 roots were significantly increased by 37.15%, 56.40%, 90.38% and 29.90%, respectively. Glycine content was significantly reduced by 69.17%. Compared with the control, the contents of aspartate, glutamic acid, cystine and phenylalanine in Z35 shoots increased by 41.63%, 21.05%, 33.70% and 58.41%, respectively, when 1.2 mmol·L^−1^ chlorinated amino acetic acid was applied. The contents of aspartic acid, glutamic acid, cystine and valine in the roots of Z35 increased by 28.02%, 22.10%, 14.86% and 33.63%, respectively, while glycine content did not change significantly.

## 4. Discussion

### 4.1. Chlorinated Amino Acetic Acid Decreased Cadmium Uptake in Rice Seedlings

Cadmium usually enters the plant in two ways—free diffusion from the intercellular space of the root epidermis or active absorption by the rice root through the symplast pathway—and then is transported to the root vascular column through the plasmodesmata [21,22]. The rice under excessive Cd stress showed chlorosis, necrosis, cell death and mineral homeostasis disorder. Rice can tolerate high cadmium through root isolation and reduced transport to the aboveground part. Excess cadmium can also be tolerated by binding to cell walls and vacuoles, as well as different compounds such as organic acids, proteins and polysaccharides [23]. The deposition of Cd in the plant root cell wall and the combination of cell wall components is the first barrier against Cd movement in the environment [24]. The presence of a large number of Cd binding sites in cell walls shows an adaptive response to Cd exposure and is a natural barrier that limits the absorption and transport of Cd into plant cells [25,26]. In this study, it was found that Cd content in subcellular fractions of rice seedlings was the highest under cadmium stress, while exogenous application of chlorinated amino acetic acid significantly reduced Cd content in the cell walls of the shoots and roots of rice seedlings.

### 4.2. Chlorinated Amino Acetic Acid Promoted the Synthesis of Ca and Mn in Rice Seedlings

At present, it is generally believed that Cd enters root cells through transporters or channels of Mn, Zn, Fe, Ca and other elements [27,28]. As an aggregation of various channel proteins, NSCCs are widely distributed in the protoplasmic membrane of plant cells and can transport plant nutrient elements such as Ca^2+^, Mg^2+^, K^+^, Zn^2+^ and Mn^2+^; they are also the main pathway for heavy metal cation Cd^2+^ to enter cells [29,30,31,32,33,34,35]. Some plant mineral elements, such as Ca, Fe and Zn, compete for the same membrane transporters as Cd. As a phytonutrient, Ca and Cd compete for the same channels in plants, which can alleviate the toxicity of Cd in plants to a certain extent [36]. Studies have shown that competition between Cd and Mn in transporters and channels plays a key role in Cd accumulation in rice organs [37]. Exogenous addition of manganese can promote plant root growth and enhance NO_3_^−^ absorption, and high Mn levels can improve plant photosynthesis by restoring chloroplast structures damaged by cadmium toxicity, thus reducing cadmium toxicity [38,39]. The present study showed that the contents of Mg, Ca, Fe and Zn in the shoots of rice seedlings were not significantly affected by exogenous application of chlorinated acetic acid, while the contents of Mn were significantly increased. Ca content in the root system was significantly increased.

### 4.3. Chlorinated Amino Acetic Acid Promotes the Synthesis of Amino Acids in Cells

Amino acids are osmotic agents that regulate ion transport, as well as precursors of small molecule antioxidants and signaling metabolites [40]. Studies have shown that amino acid metabolism in rice is also one of the important mechanisms for alleviating the toxicity of heavy metal Cd in rice [41,42,43]. By triggering amino acid synthesis in rice roots, it may reduce the toxicity of cadmium pollution in rice and reduce the bioavailability of Cd in rice plants [20]. Studies have shown that glutamate content under high Cd stress is significantly lower than that under low Cd stress [37]. Under the stress of Cd, the contents of free essential amino acids and non-essential amino acids in rice increased after the application of calcium magnesium phosphate fertilizer [43]. The plant defense system requires amino acids to synthesize different stress-responsive proteins. Aspartic acid is an important precursor of many other amino acids, which can effectively relieve plant oxidative stress under Cd stress and enhance the tolerance of rice seedlings to Cd [44]. Glutamic acid, as an important part of nitrogen-related metabolic processes, can alleviate the toxicity of Cd by forming the Glu-Cd complex [45,46]. Cysteine acts as an important precursor to the synthesis of free radical scavenger glutathione and can protect cells from oxidative damage and help alleviate plant oxidative stress induced by heavy metals [47,48]. In this study, it was found that under the stress of Cd, exogenous addition of chlorinated amino acetic acid promoted the contents of aspartic acid, glutamic acid, cystine acid and valine in the shoots and roots of rice seedlings, which could effectively alleviate the toxicity of Cd in rice seedlings.

## 5. Conclusions

The accumulation of Cd in rice leads to the damage of rice membranes and organelles and interferes with the transport and absorption of mineral elements. In this experiment, we found that under the stress of Cd, the application of 1.2 mmol·L^−1^ chlorinated amino acetic acid could effectively reduce the accumulation of cadmium in the shoots and roots of rice seedlings. The inhibition of Cd accumulation in the shoots cell wall (F1) and cytosol (F3) was much greater than that in organelles (F2). Mn content in shoots and Ca content in roots of rice seedlings increased significantly. The results of amino acid analysis showed that the application of chlorinated amino acetic acid promoted the synthesis of aspartic acid, glutamic acid and cystine in rice seedlings, which increased by 28.02%, 22.10% and 14.86%, respectively. Therefore, the application of chlorinated amino acetic acid is an effective measure to reduce the Cd concentration and increase the amino acid content of rice seedlings.

## Figures and Tables

**Table 1:** Effects of chlorinated amino acetic acid on mineral elements in X24 rice seedlings.

Treatments	T0	T1	T2	T3	T4
Elements in shoots					
K (g/kg)	49.6 ± 1.46 a	50.1 ± 2.39 a	44.6 ± 0.66 a	46.1 ± 2.47 a	47.4 ± 3.47 a
Mg (g/kg)	4.16 ± 0.14 a	4.38 ± 0.30 a	4.19 ± 0.21 a	4.25 ± 0.18 a	4.49 ± 0.39 a
Ca (g/kg)	5.53 ± 0.24 a	5.43 ± 0.30 a	5.79 ± 0.29 a	6.21 ± 0.37 a	6.20 ± 0.13 a
Fe (g/kg)	0.29 ± 0.03 a	0.22 ± 0.02 a	0.40 ± 0.03 a	0.28 ± 0.01 a	0.36 ± 0.03 a
Mn (mg/kg)	149.0 ± 0.27 b	152.2 ± 7.13 b	197.1 ± 5.13 a	198.4 ± 19.14 a	187.1 ± 7.78 ab
Zn (mg/kg)	31.8 ± 1.04 a	33.7 ± 0.43 a	32.6 ± 2.82 a	33.8 ± 0.23 a	33.1 ± 0.47 a
Elements in roots					
K (g/kg)	16.6 ± 0.61 a	12.3 ± 1.37 b	9.71 ± 1.68 bc	8.99 ± 0.18 c	13.5 ± 0.99 b
Mg (g/kg)	0.70 ± 0.06 ab	0.69 ± 0.06 ab	0.68 ± 0.03 b	0.66 ± 0.06 b	0.82 ± 0.06 a
Ca (g/kg)	2.92 ± 0.04 c	2.77 ± 0.08 c	5.17 ± 0.41 a	3.58 ± 0.22 b	3.66 ± 0.23 b
Fe (g/kg)	4.63 ± 0.39 ab	4.19 ± 0.05 b	5.36 ± 0.38 ab	6.18 ± 0.36 a	5.39 ± 0.04 ab
Mn (mg/kg)	137.8 ± 13.63 a	555.3 ± 21.63 b	624.6 ± 2.35 b	804.1 ± 16.48 a	758.9 ± 55.67 a
Zn (mg/kg)	65.3 ± 3.94 b	75.9 ± 4.47 ab	69.7 ± 4.64 ab	83.9 ± 4.97 a	62.4 ± 2.05 b

Note: T0, T1, T2, T3 and T4 respectively represent the application of 0, 0.2, 0.5, 0.8 and 1.2 mmol·L^−1^ chlorinated amino acetic acid treatment. Different lowercase letters indicate significant differences between treatments (*p* < 0.05).

**Table 2:** Effects of chlorinated amino acetic acid on mineral elements in Z35 rice seedlings.

Treatments	T0	T1	T2	T3	T4
Elements in shoots					
K (g/kg)	51.0 ± 1.54 a	46.8 ± 4.00 ab	46.7 ± 1.99 ab	44.6 ± 3.40 b	44.0 ± 3.86 b
Mg (g/kg)	4.17 ± 0.18 ab	4.61 ± 0.12 a	4.25 ± 0.17 b	4.20 ± 0.29 b	3.70 ± 0.09 b
Ca (g/kg)	6.51 ± 0.06 ab	7.25 ± 0.44 a	6.01 ± 0.01 b	6.80 ± 0.43 ab	5.74 ± 0.18 b
Fe (g/kg)	0.23 ± 0.02 ab	0.23 ± 0.02 ab	0.24 ± 0.02 a	0.22 ± 0.02 ab	0.20 ± 0.02 ab
Mn (mg/kg)	140.3 ± 12.63 bc	185.9 ± 8.18 a	150.8 ± 1.74 b	170.6 ± 10.51 a	121.7 ± 3.98 c
Zn (mg/kg)	31.0 ± 1.75 bc	31.2 ± 3.09 bc	35.7 ± 3.12 a	33.6 ± 1.85 ab	29.4 ± 1.38 c
Elements in roots					
K (g/kg)	21.9 ± 0.31 a	18.2 ± 0.83 ab	15.0 ± 1.10 b	12.6 ± 0.47 b	14.7 ± 0.75 b
Mg (g/kg)	0.82 ± 0.04 a	0.82 ± 0.03 a	0.68 ± 0.01 b	0.70 ± 0.01 ab	0.78 ± 0.01 ab
Ca(g/kg)	2.36 ± 0.13 c	2.76 ± 0.24 bc	2.74 ± 0.09 ab	3.41 ± 0.18 ab	3.45 ± 0.25 a
Fe (g/kg)	3.91 ± 0.34 b	4.97 ± 0.34 ab	6.72 ± 0.18 a	6.90 ± 0.07 ab	6.20 ± 0.09 a
Mn (mg/kg)	145.3 ± 7.45 d	785.5 ± 35.69 bc	982.4 ± 51.55 a	936.4 ± 72.72	962.0 ± 33.99 ab
Zn (mg/kg)	64.5 ± 5.60 d	74.3 ± 3.02 c	90.6 ± 2.20 a	82.2 ± 3.73 b	59.7 ± 3.24 d

Note: T0, T1, T2, T3 and T4 respectively represent the application of 0, 0.2, 0.5, 0.8 and 1.2 mmol·L^−1^ chlorinated amino acetic acid treatment. Different lowercase letters indicate significant differences between treatments (*p* < 0.05).

## Data Availability

Not applicable.
